# Dyadic Experiences of Living With Cognitive Impairment Through a 3-Year Longitudinal Qualitative Study

**DOI:** 10.1093/geroni/igaa057.2706

**Published:** 2020-12-16

**Authors:** Jing Wang, Bei Wu, Eleanor McConnell, Ding Ding, Kirsten Corazzini

**Affiliations:** 1 Fudan University, Nantong, Shanghai, China; 2 New York University, New York, New York, United States; 3 Duke University Medical Center, Durham, North Carolina, United States; 4 Fudan University Affiliated Huashan Hospital, Shanghai, China; 5 University of Maryland, Baltimore, Maryland, United States

## Abstract

The fastest growth of population living with cognitive impairment takes place in China. The estimated prevalence of cognitive impairment among older adults in China is between 13% and 20%. This study focused on persons with cognitive impairment (PWCI) and their spousal care partners to explore how spousal relationships impact dyadic experiences of living with cognitive impairment through a person-centered care lens. We conducted a longitudinal qualitative study of 10 dyads of PWCI and their care partners over three years with three data collection time points. Our findings suggest that the complexity of changing experience of living with cognitive impairment is interpreted in the dynamic nature of their spousal relationship and relationship with others, patterns of communication, daily activities and care during the extended period of cognitive decline. It is crucial to help them nurture the belief that there is a significant meaning in the journey of living with cognitive impairment.

